# Bund katholischer Ärzte: Homeopathic Conversion Therapists in 21^st^ Century Germany

**DOI:** 10.3389/fsoc.2021.667772

**Published:** 2022-01-17

**Authors:** Yannick Borkens

**Affiliations:** College of Public Health, Medical and Veterinary Science, James Cook University, Townsville, QLD, Australia

**Keywords:** Bund katholischer Ärzte, conversion therapy, Germany, homeopathy, homosexuality

## Abstract

Even in modern Germany of the 21st century there is still homophobia and other intolerances towards different sexualities and genders. These are also evident in the presence of so-called conversion therapies, which are still offered although there are already legal efforts. Among those groups, the *Bund katholischer* Ä*rzte* (Association of Catholic Doctors) is a unique curiosity. Although this group is no longer really active, it is currently moving into the German focus again due to criminal charges and reporting in the tabloid press. The aim of this publication is to bring the *Bund katholischer* Ä*rzte* not only into a more scientific but also into a more international focus. Furthermore, it is an ideal example to show what strange effects homophobia can produce.

## Introduction

When someone moves around in modern Germany, one believes to be in a liberal and cosmopolitan country. And although this is generally true, there are also movements and groups in Germany that are not only more backward, but also pursue very absurd and strange attitudes. One example is the *Bund katholischer Ärzte*, which can be translated as *Association of Catholic Doctors* or *Federation of Catholic Doctors*. Although this group was more active in the 2010s, it is currently coming back into focus thanks to various criminal charges, including from the Green politician Ulle Shauws (Bündnis 90/Die Grünen), as well as reports that have been published in some tabloid magazines (Focus online, 2020; Schenk, 2020). Since the *Bund katholischer Ärzte* (short *BkÄ*) represents ethically and morally problematic views, the reporting should not only be left to illustrated and tabloid publications, but also bring it into a scientific and international focus.

## The Disease Homosexuality, Conversion Therapies and the Current Legal Situation in Germany

Before focusing especially on groups such as the *BkÄ*, the general background on conversion therapies and the situation in Germany should be clarified. In the 21st century, homosexuality is no longer considered as a disease. One of the major classifications for diseases is the *International Classification of Diseases* (ICD), published by the *World Health Organization* (WHO). The WHO deleted homosexuality from the ICD in the year 1990, after the 43rd World Health Assembly. Since the 10th revision (ICD-10), it states that sexual orientation in itself cannot be viewed as a disorder (Cochran et al., 2014). From the 11th revision (ICD-11), this also applies to transsexuality. The term *transsexualism* is now replaced with *gender incongruence*. The ICD-11 will come into force on January 1st, 2022 (Atienza-Macas, 2020). The *American Psychiatric Association* already removed homosexuality from its *Diagnostic and Statistical Manual of Mental Disorders* in 1973 (Spitzer, 1981). Nevertheless, conversion therapies remain a problem. These therapies, also known as reparative therapies, aim at reducing homosexual tendencies and developing heterosexual potentials. Such forms of therapy are rejected in modern psychology because they contradict modern science. In Germany, since December 2019, there have been stronger efforts to ban conversion therapies through a law. This law, implemented by the *Bundesgesundheitsministerium* (Ministry of Health) and the incumbent Health Minister Jens Spahn (Merkel IV cabinet) came into force in June 2020. The law does not only focus on medical and other interventions that are intended to specifically change or suppress the sexual orientation or the self-perceived gender identity of a person, but also on promoting them. In the future, violations will result in fines up to 30,000 Euro (for violations of the promotion ban) and imprisonment of up to one year (for violations of the ban of treatment). However, the law only applies to minors (under 18 years of age) and adults whose consent is based on a lack of will (Bundesministerium für Gesundheit, 2020). In this way, dangerous loopholes as well as grey areas could arise and associations like the *BkÄ* can make use of them.

Similar laws and efforts exist in other states as well. However, a real ban on conversion therapies exists in only five countries: Germany, Malta, Brazil, Ecuador and Taiwan. In June 2021, all parties of the Austrian parliament voted in favor of a law to ban conversion therapies for minors. It is therefore very likely that Austria will be the next country in the list of countries with a ban on conversion therapies (Krenn, 2021). Canada has been negotiating its draft legislation since October 2020. Five new criminal offences are proposed in it:

- Causing a minor to undergo conversion therapy
- Removing a minor from Canada to undergo conversion therapy abroad
- Causing a person to undergo conversion therapy against their will
- Profiting from providing conversion therapy
- Advertising an offer to provide conversion therapy

**FIGURE 1:**
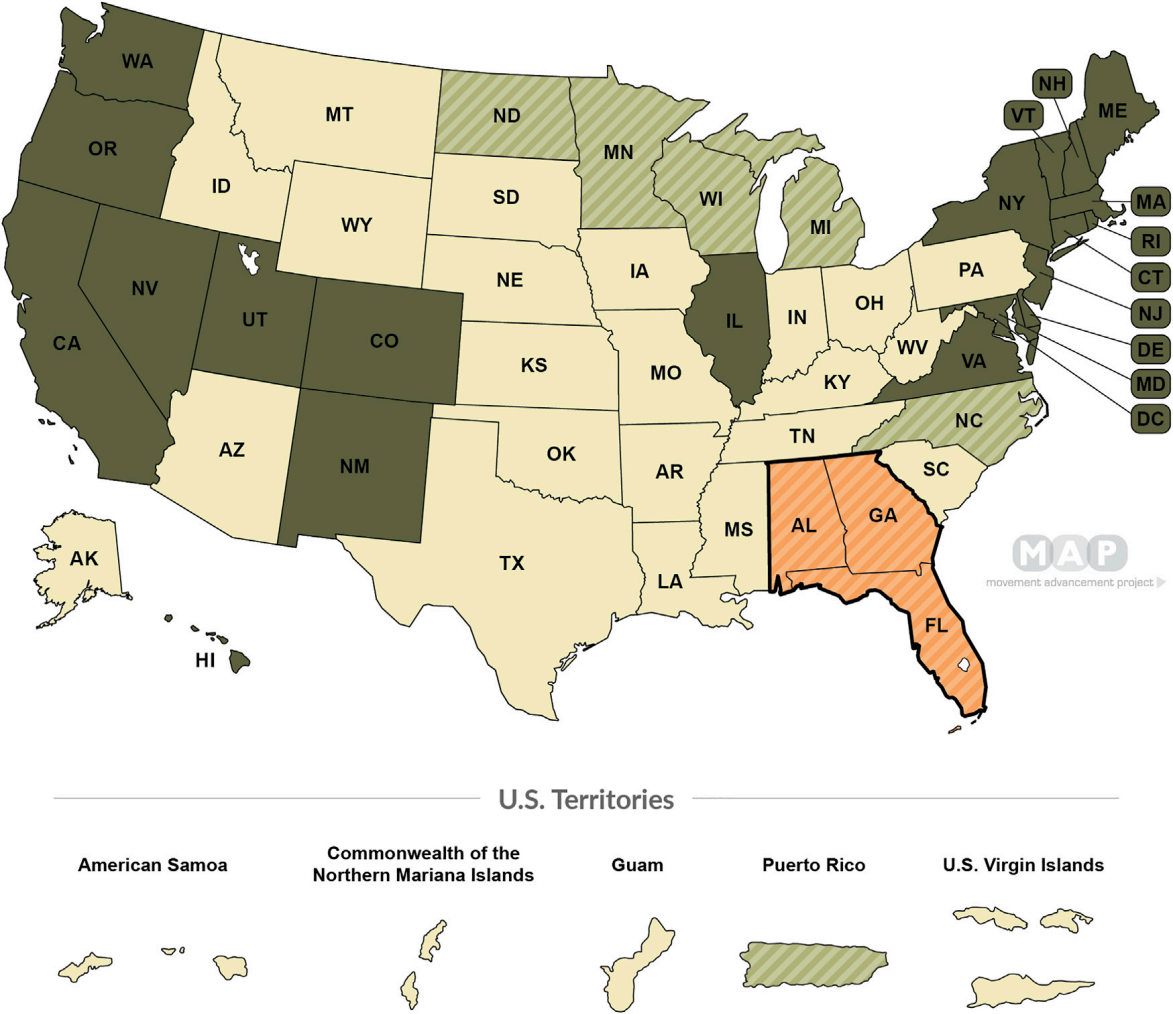
The figure shows the states and territories of the United States and their current status of their law against conversion therapies. Dark Green: State law bans conversion therapy for minors; Green Stripes: State partially bans conversion therapy for minors; Light Green: No state law or policy; Red Stripes: State is in federal judicial circuit with a preliminary injunction currently preventing enforcement of conversion therapy bans. The map comes from the Movement Advancement Project (MAP), a think tank founded in 2006 to help promote equality and opportunity for all through in-depth research, insight and communication. The data shown here is from April 10, 2021 (Movement Advancement Project, 2021).

However, further efforts by Canada have currently been put on hold (Department of Justice Canada, 2020; Reuters, 2021). Due to federalism, different laws may also apply in individual countries. In Australia, for example, only Queensland, Victoria and the Australian Capital Territory, have laws against conversion therapies. The Victoria law is the most recent of the three (Keck, 2021). The situation in the United States is complex. 20 states (and the District of Columbia) have laws similar to the one in Germany, i.e., a ban for minors. Partial bans on conversion therapy for minors exist in 5 states as well as in Puerto Rico, a territory of the United States. The majority of states (22 plus the remaining 4 territories) have neither a law nor policy at all. Alabama, Florida and Georgia are currently special cases, as these states are in federal court proceedings with an injunction currently preventing enforcement of the conversion therapy ban. Figure 1 shows the current situation in the United States. Although a majority of states in the United States do not have laws against conversion therapies, the majority of those affected, members of the LGBTQ community, live in states that have completely banned conversion therapy for minors. 48% of the LGBTQ population lives in these states. 9% in turn lives in states with a partial ban. 11% live in Alabama, Florida or Georgia. However, the population in states without any laws is still 32% (Movement Advancement Project, 2021). Currently, there are no known convictions under the new conversion therapy ban in Germany. In Malta, whose law was passed at the end of 2016, there have also been no convictions to date (Rudolph, 2019).

## Bund Katholischer Ärzte—Homeopathic Conversion Therapists in Bavaria

The *Bund katholischer Ärzte* was founded in the year 2004 as the *katholische Ärztevereinigung* (catholic medical association). In 2010, the name was changed to *BkÄ—Bund katholischer Ärzte*. The *BkÄ* was founded by Dr. (I) Gero Winkelmann, a general practitioner and homeopath based in Unterhaching (the second largest municipality in the district of Munich—Bavaria) (Winkelmann, 2013). On its website, the *BkÄ* describes its mission to bringing together people who claim to be Roman Catholic and to bringing them back to faith (Winkelmann, 2020c). According to its own information, the *BkÄ* addresses Catholic doctors and dentists, professors, medical students and employees of the medical faculties as well as priests, pastors and pharmacists or pharmacy students (Winkelmann, 2012). The *BkÄ* is said to have up to 400 members today (Psiram, 2019). However, this information is not secured. Partly because the *BkÄ* itself published little and often contradicting information in the past. Neither the exact number of members is known, nor is there any other known board members besides Gero Winkelmann (Kathpedia, 2014). There is also no development of further local *BkÄ*-groups outside of Munich and only two doctors have answered on published announcements (Winkelmann, 2005; Bund katholischer Ärzte, 2020).

Nowadays, the *BkÄ* is best known for its conversion therapies and its general homophobic attitude. On its website, the *BkÄ* describes these topics as special forms of therapy from a Catholic medical point of view (“allgemeine wie auch besondere Therapieformen aus katholischer-ärztlicher Sicht”) and to the extent to which certain forms of therapy are harmful or even acceptable to Christians (“[…] inwiefern gewisse Therapieformen schädlich oder für Christen überhaupt annehmbar sind”). The term therapies (“Therapieformen”) does not actually describe medical therapies, but rather actions, measures, character traits and sexualities like homosexuality. Other topics addressed by the *BkÄ* are abortions, the prohibition of contraceptives (condoms and birth control pills), but also medical and biologically ethical topics such as stem cell research (Winkelmann, 2020d). However, The *BkÄ* is primarily concerned with homosexuality and conversion therapies. But these differ from the classic and well-known therapies, which are mostly psychotherapies. On his online presences, Gero Winkelmann describes not only his therapeutic approaches but also scientific backgrounds, which however do not stand up to modern scientific knowledge and can (and should) be called pseudoscientific.

## Dr. (I) Gero Winkelmann—Founder of the BkÄ

In order to understand the therapeutic approaches of the *BkÄ*, it makes sense to take a closer look at its founder Gero Winkelmann. Dr. (I) Gero Winkelmann is a German general practitioner with the additional title *Homöopathie* (homeopathy). In Germany, licensed doctors can acquire the additional title of homeopathy through further training (6 courses with 40 lessons each) and are then allowed to work and treat as homeopaths (Deutscher Zentralverein homopathischer Ärzte, 2018). However, this training is very controversial. According to his own statements, Gero Winkelmann himself uses homeopathy not only in his private practice but also in emergencies when he is on call (Winkelmann, 2020a). His actual medical degree was not obtained in Germany but in Italy, more precisely in Ancona and Verona. There he studied medicine between 1977 and 1984 and obtained the Italian degree *Dottore in Medicina e Chirurgia* (doctor of medicine and surgery). This degree was recognized by the *Kultusministerium* (Ministry of Education and cultural affairs) in North Rhine-Westphalia in 1984 and converted into the German degree *Dr. (I)* (Psiram, 2019). Nowadays he is best known for his homophobic and comparable views. He describes himself as a doctor who also practices his Catholic faith at work (Winkelmann, 2019). According to him, this is achieved through three pillars: Christian ethics, especially at the beginning and the end of life (“Christliche Ethik, insbesondere am Lebensanfang und Lebensende”), prayers and visits to church services (“Gebete und Besuch von Gottesdiensten”) and to participate in church services on public holidays and also on working holidays, especially as a doctor on call who works at *untimely* times (e.g. holidays, at night) (“Gerade als Bereitschaftsarzt, der zu “Unzeiten” tätig ist (feiertags, nachts) […] die Gottesdienste an Feiertagen und auch an Werktagen (z.B. Rorate-Amt) zu besuchen”) (Winkelmann, 2019).

The point of Christian ethics, in particular, requires a closer look. “Christian ethics, especially at the beginning and the end of life” results in a pro-life view. In the United States, this pro-life view, also known as pro-life movement, results not only in demonstrations and political petitions but also in violence, sometimes with death consequences. Since 1993, at least 11 people have been killed in attacks on abortion clinics, mostly doctors and physicians who perform abortions. The perpetrators can often be assigned to a radical Christian spectrum (Stack, 2015). In Germany, Winkelmann is both an opponent of abortion and contraceptives as well as an opponent of medical euthanasia. Before he became known throughout Germany through the establishment of the *Bk*Ä, he founded the European Pro-Life Doctors (2020) association, which campaigns against abortion, but also against contraceptives and research such as stem cell therapy. The foundation was in the year 2000. However, there is neither an official establishment of the EPLD as an official association nor an official entry in the German association register. This also applies to the *Bk*Ä (European Pro-Life Doctors, no publication date; Winkelmann, 2016b). In addition to the rejection of abortions and euthanasia, Winkelmann uses the EPLD webpage also to spread the view that condoms do not work and also do not help against AIDS. He explains this with the fact that the latex skin of the condom is too thin and the HI-Virus could penetrate it without any problems. According to him, this made condoms reflect a pseudo-security (Winkelmann, 2016a). Furthermore, he also defended the statement of Pope Benedict XVI, that condoms could not help against AIDS. Pope Benedict XVI stated that on his Africa trip in 2009 (Welle, 2009). Especially for men who have sex with men (MSM), the condom is an important means of protection against AIDS infections (Wirth et al., 2020). This is especially true in African countries that have a high AIDS rate. In some parts, the AIDS rate is 100 times higher than in the United States (with a similar sexual activity) (Epstein and Ashburn, 2004).

On his website, Winkelmann not only describes the scientific background of homosexuality and his conversion therapy (which, however, does not stand up to modern scientific findings) but also defends the use of conversion therapies in general. He describes that every bad state needs a reversal (conversion). Furthermore, users of those therapies should not be afraid of a reversal, inner maturation and strengthening of self-healing powers. In addition, users should not let themselves be deterred from improving the situation. Those statements prove that Winkelmann considers homosexuality to be an evil condition that requires conversion, which in turn leads to inner maturation. According to Winkelmann, there are various causes for homosexuality. The causes include hormones, liver damages, epigenetically transmitted syphilis or abuse in childhood (Lau, 2018; Psiram, 2019). Thus, he contradicts not only biological-medical but also socio-behavioral knowledge. However, his conversion therapies differ from the classic conversion therapies, which are often psychotherapies. In contrast to these, Winkelmann uses homeopathy in his conversion therapies. He himself does not call his therapy conversion therapy but constitution therapy. This constitution therapy is a whole-body therapy that is primarily intended to stimulate physical and emotional self-healing. In that therapy, he combines homeopathy with psychotherapy and religious care. At the beginning, the body is detoxified with sulfur globules and nosodes, globules made from pathological material. Winkelmann says that the therapy has already been successfully completed for many after this detoxification. It should be noted that there is no information available on the number of people who accepted Winkelmann’s offer. If the detoxification is not enough, a lengthy homeopathic therapy begins, which also includes psychological and religious support. In this therapy, Winkelmann uses Calcium Carbonicum and Clacium Phosphoricum globules. Religious support includes prayers, sacraments, anointing of the sick and the holy communion (Lau, 2018).

## Short Introduction About Homeopathy

Homeopathy is the most common alternative medicine. It was developed by the German doctor Samuel Hahnemann. Nowadays it is regularly criticized as a medical method and is part of medical as well as philosophical discourses. These discourses are primarily driven by the fact that homeopathy both contradicts the known laws of nature and is generally unscientific (Grams and Endruscheit, 2020b). Since it was invented 200 years ago, there has been no evidence of its effectiveness. Many published studies that describe a positive effect have glaring errors, for example in study design (Linde, 1999; Ernst, 2011; Schmacke, 2020; Walach, 2020). Nevertheless, homeopathy is very popular. The reasons for this are not only the successful work of lobbyists but also relatively uncritical reporting in the mass media (Harder, 2020a; Grams and Endruscheit, 2020a). Winkelmann uses homeopathy not only in his conversion therapies but also for serious medical complications and diseases. Furthermore, he promotes homeopathy as a cure for COVID-19. He is referring to a report from India, according to which homeopathy was allegedly used successfully against COVID-19 in India (Winkelmann, 2020b). This report was refuted by the Indian ministry of health shortly after it was published (Feldwisch-Drentrup, 2020; Scholz, 2020). However, alternative healing methods such as homeopathy remain a serious threat during the COVID-19 pandemic. It is assumed that these alternatives have so far cost the lives of several hundred people. Further damages are poisoning (caused by the consumption of disinfectants or high percentage alcohol) or blindness (Harder 2020b; Islam et al., 2020). In addition to homeopathy, the touted remedies include cow urine, bleach or cocaine (Caulfield, 2020). This problem has become so serious that scientists are now talking about an *Infodemic*, the first in human history (Caulfield, 2020; Chakrabarthi and Narayan, 2020; Sánchez Tarragó, 2020). Scientists need to respond to this new threat.

## Conclusion

Even today, homophobia is a relevant topic that still affects the lives of many homosexuals. Many homosexual men and women have been subjected to constant discrimination and stigma while trying to do what most heterosexual individuals do. This includes not only things like getting married, but also other things like serving in the military or in professional sports (Sinclair, 2009; Luisi et al., 2016; Ofosu et al., 2019; Brickell, 2020). Outings of well-known United States athletes generated a great amount of media attention and questions about the defining of masculinity (Luisi et al., 2016). But homophobia and stigma are also still relevant in medicine. Intersexuals, for example, are also still sometimes viewed as deviations in need of treatment (Hester, 2004). Another critical and important aspect of modern medicine is the persistent failure of research instruments to account for gender differences in study design and treatment (Holdcroft, 2007). In order to create a liberal and tolerant medicine, problems like these need to be addressed. It is not only important to identify pseudomedicine and false information as such, but also to educate patients and medical laypersons about them. In doing so, it is important not to dismiss doctors and other people like Winkelmann simply as weirdos or the like (as many scientists still do), but to take them seriously. Studies have already shown that information and education help to reduce not only fear but also stigma and other things like homophobia and racism (Paradies, 2005; Waszak et al., 2018; Ofosu et al., 2019). Furthermore, the discussion about the *BkÄ* also arises the question of how closely religion and religious institutes (represented here by the Christianity) should be linked to modern medicine and its infrastructure. The *BkÄ* has meanwhile distanced itself from its conversion therapy (Queer.de, 2020). However, it is questionable how seriously this step ultimately is.

## Data Availability

The original contributions presented in the study are included in the article/Supplementary Material, further inquiries can be directed to the corresponding author.
